# Cooling and heating season performance of an open counterflow heat-source tower heat pump system in high-humidity climates: An experimental and numerical study

**DOI:** 10.1371/journal.pone.0337196

**Published:** 2026-02-17

**Authors:** Xinhao Liu, Peng Liu, Dinggao Xiao, Yuan Li, Shuangying Yang

**Affiliations:** 1 School of Civil Engineering, Guizhou University, Guiyang, China; 2 Guiyang Architectural Design and Surveying Prospecting Co., Ltd., Guiyang, China; University of Shanghai for Science and Technology, CHINA

## Abstract

The cooling and heating season performance of an open counterflow heat-source tower heat pump (HTHP) system in high-humidity climates is investigated through an integrated experimental and numerical study, focusing on its year-round operation under typical conditions. The heat and mass transfer models for summer and winter were validated using measured data. Results show that increasing the inlet air temperature in summer enhances latent heat transfer by 25% but reduces sensible heat transfer by 31%. Raising the water temperature from 28.0 °C to 34.0 °C resulted in a 20.7% increase in latent heat transfer and a 16.7% decrease in sensible heat transfer, thereby increasing the latent heat ratio to 87.19%. In winter, increasing the inlet air temperature from −1.0 °C to 7.0 °C enhanced sensible but reduced latent heat transfer. Higher solution temperatures reduced total heat transfer, and the latent heat ratio dropped to 18.2%. The inlet air humidity ratio had a greater effect on sensible heat transfer in summer, and the opposite in winter. Increasing the inlet solution mass flow rate enhanced the total heat transfer in both seasons, while the latent heat ratio slightly rose. Operational tests demonstrated that the system achieved *COP* ranging from 2.43 to 3.23 under typical January conditions and 3.8 to 5.0 under typical June conditions. The performance evaluation yielded a Heating Seasonal Performance Factor (*HSPF*) of 3.06, a Seasonal Energy Efficiency Ratio (*SEER*) of 4.3, and an Annual Performance Factor (*APF*) of 3.62. These results confirm the system’s favorable year-round performance and offer guidance for HTHP applications in Guizhou and similar high-humidity regions.

## 1. Introduction

Optimizing energy efficiency in air conditioning systems plays a key role in fostering low-carbon, sustainable buildings. According to the 2024 report on carbon emissions from China’s urban and rural building sector [1], about 22% of national final energy was consumed by buildings in 2022, and the building sector accounted for 21.7% of energy-related carbon emissions. Within the building sector, approximately 60% of energy use was for space cooling and heating. Therefore, the development of reliable and efficient thermal energy systems is crucial for lowering energy consumption and emission levels in the building industry [2]. Among existing building thermal systems, chillers and cooling towers exhibit high efficiency and energy savings but require ample installation space [3,4]. Air-source heat pumps are the most widely used systems owing to their flexibility, compact size, energy efficiency, and environmentally friendly operation [5]. However, they are prone to frost under cold and humid winter conditions. Frequent defrosting is required to maintain regular operation [6], which increases energy use and reduces heating capacity [7]. Ground-source heat pumps utilize shallow geothermal energy, offering high efficiency and significant savings. However, their application is limited by geological conditions [8].

The heat-source tower functions as a device for upgrading low-grade thermal energy.

In winter, the low-enthalpy circulating solution absorbs low-grade thermal energy from humid cold air with higher enthalpy. In summer, intensive thermal and mass exchange occur between high-enthalpy circulating water and low-enthalpy air, transferring low-grade thermal energy to high-grade thermal energy. The system delivers cooling in summer and heating in winter, and supplies hot water throughout the year, thereby improving overall equipment utilization. In winter, the antifreeze solution lowers the freezing point of the liquid to below −10 °C. This design mitigates the frosting problem of air-source heat pumps in cold climates and circumvents the geographical and geological constraints of ground-source heat pumps. Based on whether the circulating medium is in direct contact with air, heat-source towers can be categorized into two types: open and closed. Open heat-source towers enable simultaneous exchange of sensible and latent heat. Although their construction costs are higher than those of closed types, they provide superior heat transfer efficiency. According to the relative flow direction of the circulating medium and air, heat-source towers are classified into two types: crossflow and counterflow. In counterflow heat-source towers, air flows upward through the packing, whereas in crossflow types, air flows horizontally through the packing. Counterflow towers entail higher maintenance costs but achieve greater heat transfer efficiency than crossflow towers.

Many scholars have extensively studied the heat and mass transfer modeling and practical applications of heat-source tower heat pump systems. The theoretical model of the open heat source tower is developed based on Merkel’s model for cooling towers [9]. However, the model did not account for the effect of evaporation [10] or local air flow distribution [11]. Liu et al. [12] developed numerical models for heat-source tower and air-source heat pump systems, based on which they suggested adaptations for varied climatic zones. Lv et al. [13] introduced a variable Lewis number to enhance accuracy, and Huang et al. [14–16] proposed an enthalpy-difference-driven model for both summer and winter operation. Furthermore, simulation studies proposed a performance comparison method between heat-source and air-source heat pumps, demonstrating that the HTHP system exhibits superior energy efficiency.

Zendehboudi et al. [17] employed ethylene glycol as the working fluid to conduct experimental studies on a closed crossflow tower under cold conditions, and performed a comprehensive sensitivity analysis of the associated input parameters. Huang et al. [18] used calcium chloride solution as the working fluid. By adjusting the operating parameters, they determined the optimal liquid-to-gas ratio and analyzed the trends in latent heat transfer. Song et al. [19] used calcium chloride and glycerol as working fluids to examine the effects of inlet parameters on the air and solution sides of closed heat-source towers. They further established correlations for heat and mass transfer coefficients. Cui et al. [20–22] performed experiments on counterflow heat-source towers with calcium chloride as the working fluid. They replaced the conventional gravity-fed liquid distribution with upward and downward spray modes. The results showed that this approach enhanced the heating efficiency. Li et al. [23] proposed a composite enthalpy-enhanced HTHP system that utilizes a non-toxic and environmentally friendly potassium formate solution as the antifreeze fluid, thereby reducing both the corrosion risk and regeneration energy consumption compared to conventional systems. Xiao et al. [24] proposed a self-heat-storage heat pump system that recovered excess heat from a heat-source tower to address the drawbacks of existing solution regeneration technologies, including additional heat demand and low efficiency. They examined the system’s performance under three heating modes, but its applicability in low-temperature conditions still requires further study. Yang et al. [25] evaluated the heating performance of open counterflow heat-source towers using actual engineering cases. Under low-temperature, high-humidity conditions, the coefficients of performance (*COP*) ranged from 2.18 to 2.94, with a seasonal performance factor of 3.18. Liu et al. [26] developed a numerical model for an open counterflow heat-source tower. They analyzed the impact of operational and environmental parameters on the tower’s heating efficiency during winter operation. A comparative analysis of energy efficiency and economic viability was conducted between the HTHP and air-source heat pump systems. Results indicate that the HTHP system demonstrates more stable heating capacity, higher efficiency, and lower operating costs under low-temperature, high-humidity conditions.

In previous research on HTHP systems, most studies have focused on the heat and mass transfer mechanisms in winter and operational optimization under winter conditions. However, a unified framework that systematically covers both cooling and heating seasons remains lacking, particularly for applications in high-humidity climates. Specifically, the cooling energy efficiency in summer and comprehensive year-round performance under typical seasonal operating conditions have yet to be thoroughly assessed and supported by data. To address this gap, this study presents a systematic analysis of an open counterflow HTHP system in high-humidity climates through integrated experimental and numerical investigations, aiming to provide engineering insights and technical guidance. The main contributions of this study are as follows:

(1) Development and validation of a mathematical model: A mathematical model has been developed to simulate the heat and mass transfer processes of the open counterflow heat-source tower during both winter and summer seasons, with validation against experimental data. This model provides the theoretical foundation for the analysis of cooling and heating energy efficiency.
(2) Analysis of seasonal heat and mass transfer characteristics: The heat transfer performance of the HTHP system has been studied for both summer and winter seasons, elucidating the seasonal differences in heat and mass transfer mechanisms. The study also investigates how inlet operating parameters influence system performance differently in summer and winter.
(3) Provision of performance metrics under typical seasonal conditions: Including Coefficient of Performance (*COP*), Seasonal Energy Efficiency Ratio (*SEER*) for the cooling season, Heating Seasonal Performance Factor (*HSPF*) for the heating season, and Annual Performance Factor (*APF*), offering references for system design and performance evaluation in regions with similar climatic conditions.

## 2. Model development

### 2.1. Assumptions of the physical model

**Fig 1:**
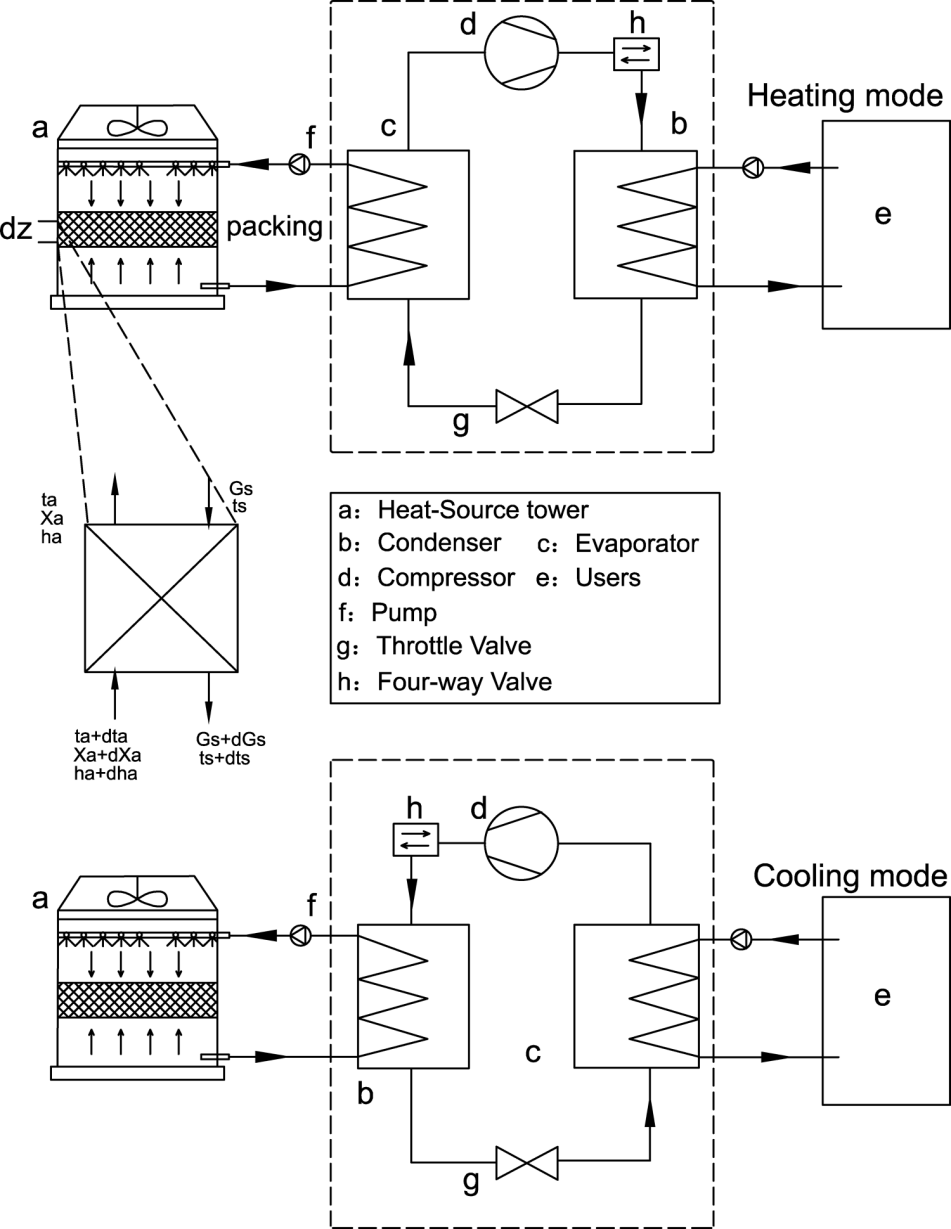
System flow diagram.

The HTHP system consists of an open counterflow heat-source tower, heat pump units, a four-way valve, heat-source-side circulation pumps, and user-side circulation pumps. Fig 1 illustrates the system’s flow diagram, with the heating mode at the top and the cooling mode at the bottom. The four-way valve is the key component for mode switching, achieved by reversing the refrigerant flow, as detailed in Fig 1 and summarized in Table 1.

**Table 1:** Four-way valve operation in heating and cooling modes.

Mode	Compressor discharge connection	Compressor suction connection	Four-way valve operation
Heating mode	Compressor → Valve → User-side Heat Exchanger (as Condenser)	Source-side Heat Exchanger →Valve → Compressor (as Evaporator)	The four-way valve directs flow from the compressor’s high-pressure side to the user-side heat exchanger, releasing heat.
Cooling mode	Compressor →Valve → Source-side Heat Exchanger (as Condenser)	User-side Heat Exchanger →Valve → Compressor (as Evaporator)	The four-way valve switches flow, the system absorbs heat from the user-side and releases it to the environment through the source-side heat exchanger and the heat-source tower.

In the open counterflow heat-source tower investigated in this study, the circulating solution flows vertically downward from the top of the packing. It drains through the packing layer under gravity. Air enters the packing layer at the bottom and rises upward. The circulating solution and air are in direct contact, enabling counterflow heat exchange. This mechanism fundamentally differs from that in closed-type heat-source towers, where heat is transferred indirectly through solid tube walls that separate the fluid and the air.

The theoretical model of the open counterflow heat-source tower is established under the following assumptions:

(a) The heat and mass transfer processes are assumed to occur exclusively on the packing surfaces within the heat-source tower, which is considered adiabatic and impermeable to moisture with respect to the external environment.
(b) The inlet moist air and circulating solution are assumed to be uniformly distributed over the packing, with stable flow rates, and always remaining in thermodynamic equilibrium.
(c) At any vertical cross-section along the airflow direction, the air’s and circulating solution’s physical properties within the tower are assumed uniform and vary only in the vertical direction.
(d) The packing is assumed to have uniform and stable physical properties, and the heat and mass transfer areas are identical.
(e) The specific heat at constant pressure and kinematic viscosity of calcium chloride solution, dry air, water vapor, and water are assumed to remain constant throughout the process.

### 2.2 Mathematical model under winter conditions

The partial pressure difference between water vapor and the solution surface drives mass transfer. Based on mass conservation, the increase in solution mass flow rate resulting from mass exchange with moist air inside the heat-source tower can be expressed as:


$$dG_{s}=h_{m}\left(X_{a}-X_{s}\right)A$$ (1)

Where $dG_{s}$ is the increase in solution mass flow rate, kg/s; $h_{m}\;$ is the mass transfer coefficient, kg/(m²·s); $X_{a}$ is the humidity ratio of the main air stream, $X_{s}$ is the saturated

humidity ratio of air at the solution surface, kg/kg; A is the gas-liquid contact area, m².

The air-side water vapor balance equation, derived from mass conservation, is expressed as:


$$-G_{da}\cdot dX_{a}=h_{m}\left(X_{a}-X_{s}\right)A$$ (2)

Where $\;G_{da}$ is the dry-air mass flow rate, kg/s; $dX_{a}$ is the reduction in moist-air humidity ratio, kg/kg.

The inlet and outlet air enthalpies can be expressed as:


$$h_{a,i}=C_{pa}t_{a,i}+X_{a,i}\left(C_{pv}t_{a,i}+r_{o}\right)$$ (3)

$$h_{a,o}=C_{pa}t_{a,o}+X_{a,o}\left(C_{pv}t_{a,o}+r_{o}\right)$$ (4)

Where $C_{pa}$ and $C_{pv}$ are the specific heat at constant dry air and water vapor pressure, J/(kg·K). $r_{o}$ is the latent heat of vaporization of water at 0 °C, kJ/kg.

Based on energy conservation, the energy balance equation of the air–solution system is formulated as Equation (5). The left-hand side denotes the heat released by heat and mass transfer, while the right-hand side includes the convective heat transfer induced by temperature differences and the latent heat released from vapor condensation in humid air.


$$G_{da}\left(h_{a,i}-h_{a,o}\right)=h_{c}\left(t_{a}-t_{s}\right)A+G_{da}\cdot dX_{a}\left(C_{pv}-t_{s,i}+r_{o}\right)$$ (5)

Where $h_{c}$ is the heat transfer coefficient, W/(m²·K); $t_{s}$ is the solution temperature, $\;t_{a}$ is the air temperature, °C.

Equation (5) simplifies to Equation (6):


$$\left(G_{da}C_{pa}t_{a,i}+C_{pv}G_{da}X_{a,i}t_{a,i}\right)-\left(G_{da}C_{pa}t_{a,o}+C_{pv}G_{da}X_{a,o}t_{a,o}\right)=C_{pv}G_{da}dX_{a}t_{s,i}+h_{c}\left(X_{a}-X_{s}\right)A$$ (6)

Likewise, the energy equation on the solution side can be written in terms of the enthalpy change of the solution as:


$$G_{s,o}C_{ps}t_{s,o}-G_{s,i}C_{ps}t_{s,i}=dG_{s}\left(C_{pv}t_{s,i}+r_{o}\right)+h_{c}\left(t_{a}-t_{s}\right)A$$ (7)

After rearrangement, it can be written as:


$$G_{s,o}C_{ps}t_{s,o}-G_{s,i}C_{ps}t_{s,i}=\left(G_{s,o}-G_{s,i}\right)\left(C_{pv}t_{s,i}+r_{o}\right)+h_{c}\left(t_{a}-t_{s}\right)A$$ (8)

Where $C_{ps}$ is the specific heat at constant pressure of calcium chloride solution, J/(kg·K).

Equations (1), (2), (6), and (8) constitute the mathematical model of heat and mass transfer between air and calcium chloride solution in the open counterflow heat-source tower under winter operating conditions.

The contact area *A* between air and solution can be expressed as:


$$A=\frac{\beta LBW}{D_{n}}$$ (9)

Where β denotes the packing’s porosity; *L*, *B*, *W* are the packing’s length, width, and height, respectively; *D* represents the spacing between the packing elements, m; and *n* refers to the number of vertical divisions in the packing.

### 2.3. Mathematical model under summer conditions

The increase in water mass flow rate due to moisture exchange inside the tower is expressed as:


$$dG_{w}=h_{m}\left(X_{a}-X_{w}\right)A$$ (10)

Based on mass conservation, the air-side water vapor balance equation can be expressed as:


$$-G_{da}\cdot dX_{a}=h_{m}\left(X_{a}-X_{w}\right)A$$ (11)

According to energy conservation, the energy balance equation between air and water, simplified in terms of the enthalpy change of moist air at the inlet and outlet, can be written as:


$$\left(G_{da}C_{pa}t_{a,i}+C_{pv}G_{da}X_{a,i}t_{a,i}\right)-\left(G_{da}C_{pa}t_{a,o}+C_{pv}G_{da}X_{a,o}t_{a,o}\right)=C_{pv}G_{da}dX_{a}t_{w,i}+h_{c}\left(X_{a}-X_{w}\right)A$$

(12)

Similarly, according to the law of energy conservation, it can be simplified in terms of the enthalpy change of water at the inlet and outlet as:


$$C_{pw}\cdot G_{w,i}\cdot \left(t_{w,o}-t_{w,i}\right)=h_{c}\left(t_{a}-t_{w}\right)A+r_{w}\left(t_{w}\right)\cdot dG_{w}$$ (13)

Where $\;dG_{w}$ is the increase in water mass flow rate, kg/s; $X_{w}\;$ is the saturated humidity ratio of air at the water surface, kg/kg; $C_{pw}$ is the specific heat at constant pressure of water, J/(kg·K); $t_{w}$ is the water temperature, °C; $r_{w}\left(t_{w}\right)$ is the latent heat of vaporization of water at temperature $\;t_{w}$, kJ/kg.

Equations (10), (11), (12), and (13) constitute the mathematical model of heat and mass transfer between air and water in the open counterflow heat-source tower under summer operating conditions.

### 2.4. Parameter determination

#### 2.4.1. Specific heat at constant pressure.

The temperature variation of calcium chloride solution in open counterflow heat-source towers generally remains within 5 °C. The specific heat at constant pressure of calcium chloride solution exhibits slight variation and can be approximated as $\;C_{ps}$ = 3320 J/(kg·K); The specific heat at constant pressure of dry air is assumed to be $\;C_{pa}$ = 1005 J/(kg·K); The specific heat at constant pressure of water vapor is thought to be $\;C_{pv}$ = 1846 J/(kg·K); The specific heat at constant pressure of water is taken to be $\;C_{pw}$ = 4200 J/(kg·K) [27,28].

#### 2.4.2. Saturated humidity ratio of air at the surface of the solution.

According to relevant literature [27], the saturated humidity ratio of air at the surface

of the calcium chloride solution $\;X_{s}$ can be expressed as:


$$X_{s}=0.622\frac{P_{s}}{P_{o}-P_{s}}$$ (14)

Where $P_{o}$ is the atmospheric pressure is taken as 101325 Pa, and $\;P_{s}\;$, representing the saturated vapor pressure above the solution surface, is defined as [29]:


$$\mathrm{lg}P_{s}=10.055-\frac{1668.374}{t_{s}}+228.043$$ (15)

#### 2.4.3. Saturated humidity ratio of air at the surface of water and latent heat of vaporization of water.

According to relevant literature [30], the saturated humidity ratio of air at the water surface$\;X_{w}\;$ and the latent heat of vaporization released during water vapor condensation$\;r_{w}\;$ can both be approximated as single-valued functions of water temperature$\;t_{w}$.

The fitted correlation between the variables is given as:


$$X_{w}=\left[0.0144t_{w}^{4}-0.7461t_{w}^{3}+33.6887t_{w}^{2}+50.0495t_{w}+4266.8615\right]\times 10^{-3}$$ (16)

The fitted correlation between the variables is given as:


$$r_{w}=\left[-2.377t_{w}+2500.673\right]\times 10^{3}$$ (17)

#### 2.4.4. Convective heat and mass transfer coefficient.

According to relevant literature [31], the convective heat transfer coefficient in winter

$\;h_{c}\;$ can be expressed as:


$$hc=\frac{k_{a}\left(2.0+0.6Re^{\frac{\text{1}}{\text{2}}}Pr^{\frac{\text{1}}{\text{3}}}\right)}{D}$$ (18)

$$Re=\frac{D\times 10^{5}}{L\times B}\left(\frac{G_{da}+G_{da}X_{s}}{1.2}+\frac{G_{w}}{1130}\right)$$ (19)

$$Pr=\frac{v_{a}c_{pa}\rho_{a}}{k_{a}}$$ (20)

Where $\;k_{a}\;$ is the thermal conductivity of moist air, $k_{a}$ = 0.0244 W/(m²·K); *D* is the packing spacing; Re and Pr are the Reynolds number and Prandtl number; $v_{a}$ is the kinematic viscosity, m²/s. Respectively, which can be determined from Equations (19) and (20). By combining Equations (18), (19), and (20), the heat transfer coefficient can be rewritten as:


$$h_{c}=\frac{0.0488}{D}+\frac{3.676}{\left(L\times B\times D\right)0.5}{\left(\frac{G_{da}+G_{da}x_{s}}{1.2}+\frac{G_{w}}{1130}\right)}^{0.5}$$ (21)

According to relevant literature [31,32], the convective mass transfer coefficient in winter $\;h_{m}\;$ can be expressed as:


$$h_{m}=\frac{h_{c}}{\rho_{a}c_{a}}{\left(\frac{Pr}{S_{c}}\right)}^{\frac{\text{2}}{\text{3}}}$$ (22)

$$S_{C}=v_{a}/D_{v}$$ (23)

$$D_{v}=\frac{435.7{\left(t_{a}+27.15\right)}^{1.5}{\left(\frac{\text{1}}{MA}+\frac{\text{1}}{MB}\right)}^{\frac{\text{1}}{\text{2}}}}{p_{o}\left(V_{A}^{\frac{\text{1}}{\text{3}}}+V_{B}^{\frac{\text{1}}{\text{3}}}\right)}\times 10^{-4}$$ (24)

Where the Schmidt number $\;S_{C}\;$ is defined as the ratio of kinematic viscosity to diffusion coefficient; $\;D_{v}\;$ is the diffusion coefficient of water vapor; MA and MB are the molar masses of water vapor and air, respectively; $\;V_{A\;}$ and $\;V_{B\;}$ are the molar volumes of water vapor and air, respectively. By combining Equations (22), (23), and (24), the mass transfer coefficient is rewritten as:


$$h_{m}=2.754\times 10^{-6}h_{c}\left(t_{a}+273.15\right)$$ (25)

Based on relevant literature [33], the convective heat transfer coefficient in summer can be expressed as:


$$h_{c}=\frac{c_{p,ma}\cdot m_{a}\cdot (t_{a,i}-t_{a,o})}{(t_{a,av}-t_{w,av})A}$$ (26)

$$t_{a,av}=\left(t_{a,i}+t_{a,o}\right)/2$$ (27)

$$t_{w,av}=\left(t_{w,i}+t_{w,o}\right)/2$$ (28)

$$c_{p,ma}=1.005+1.842X_{a,i}$$ (29)

$$m_{a}=G_{da}\left(1+X_{a}\right)$$ (30)

Where $c_{p,ma}$ is the specific heat at constant pressure of moist air, J/(kg·K).

To account for the measurement uncertainty of the inlet air humidity ratio $X_{a,i}$, the error propagation in the calculation of the specific heat $c_{p,ma}$ was considered. Based on the measurement uncertainty of ^±^ 5% (instrument accuracy) for the humidity ratio, the propagation of error in $c_{p,ma}$ was calculated, and the resulting average error was found to be 0.001603 J/(kg·K). This indicates that the measurement uncertainty has a minimal effect on the overall calculation of $c_{p,ma}$, and the results remain reliable within the experimental accuracy.

According to relevant literature [11, 31, 33], the convective mass transfer coefficient in summer $\;h_{m}\;$ can be expressed as:


$$h_{m}=\frac{G_{w}}{k\cdot V}\int t_{w,o}t_{w,i}\frac{c_{pw}}{h^{\prime \prime }-h}dt$$ (31)

$$k=1-\frac{c_{pw}\cdot t_{w,o}}{586-0.56\left(t_{w,o}-20\right)}$$ (32)

$$h^{\prime \prime }=1.01t_{w}+X_{w}\left(2500+1.84t_{w}\right)$$ (33)

$$h=1.01ta+X_{a}\left(2500+1.84ta\right)$$ (34)

### 2.5. Numerical solution

The computational model is developed using the finite difference method, in which the continuous problem is discretized into a set of difference equations. According to the preceding assumptions, at any vertical cross-section along the airflow direction, the physical properties of the air and the circulating solution inside the tower are assumed to be uniform, varying only in the vertical direction. In the two-dimensional computational domain, the packing is subdivided into n vertical segments to simulate heat and mass transfer between the air and the circulating solution within the packing.

The iterative solution within the computational domain can be described as follows:

Step 1: For each segment in the computational domain, a set of input parameters

is defined, including the enthalpy of the solution and air, the solution mass flow rate, the humidity ratio of air, and the solution concentration.

Step 2: The i-th segment is selected as the computational unit.

Step 3: The output parameters of the i-th segment are assigned as the input parameters of the (i + 1)-th segment.

Step 4: The procedure is repeated iteratively until the n-th segment is reached.

Step 5: Upon completion, the outlet state parameter distributions of the solution and air distributions are obtained.

**Fig 2:**
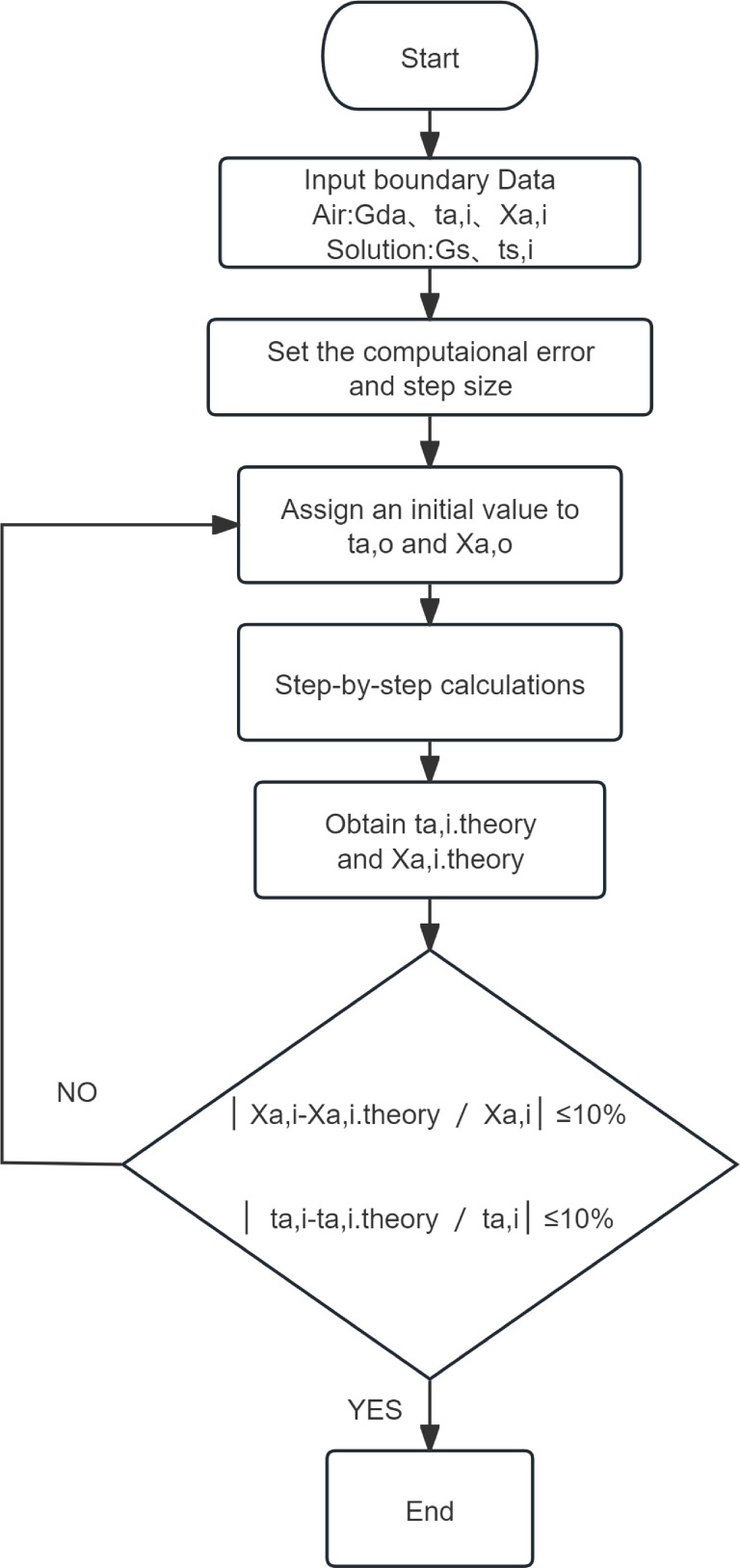
Solution flow diagram for winter operating conditions.

According to previous studies [34], dividing the packing of the counterflow heat-source towers into 10–20 computational segments vertically can satisfy calculation accuracy requirements. In the numerical solution of present research, the packing of the tower is subdivided into 10 vertical segments, i.e., n = 10. Fig 2 presents the solution flow diagram for winter operating conditions; for summer conditions, the subscript s is replaced by w.

## 3. Model validation

### 3.1. Field measurements

**Fig 3:**
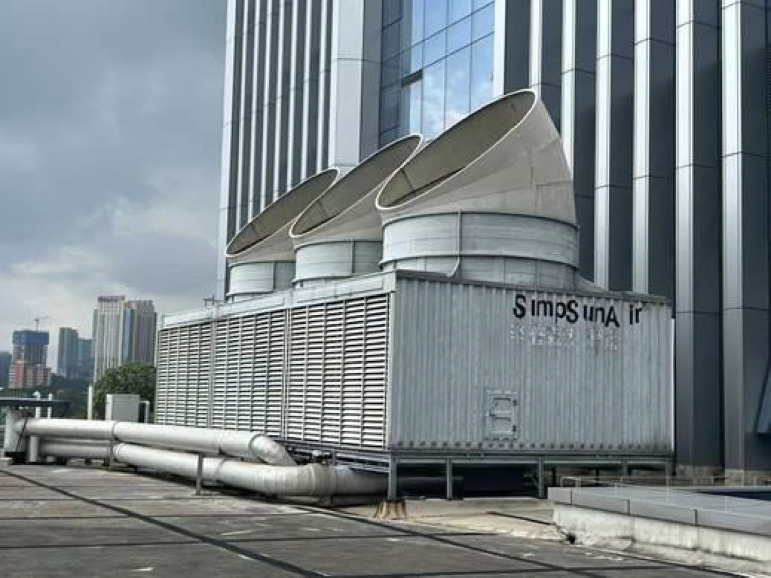
Photograph of the open counterflow heat-source tower.

The field test was conducted in a comprehensive office building in Guiyang City, Guizhou Province. The heat-source tower has dimensions of 4,200 mm × 6,300 mm × 5,400 mm, with polyvinyl chloride (PVC) packing that provides a specific surface area of 300 m²/m³. Water was used as the circulating medium during summer, while a calcium chloride solution was used in winter. The heat-source tower is shown in Fig 3. A one-year monitoring program was implemented to better evaluate the operational performance of the HTHP system. The cooling season extended from May 24 to September 20, 2023, whereas the heating season lasted from November 17, 2023, to March 15, 2024.

Field tests were carried out on representative climate days in both summer and winter. The summer test was conducted from 12:15–14:55 on June 29, 2023, and the winter test was conducted from 11:30–14:30 on December 18, 2023. To ensure the validity of the data, the test periods were deliberately scheduled to avoid local thunderstorms and other transient weather disturbances. The subsequent data selection followed two key principles: (1) Continuity: only data from uninterrupted, stable system operation were considered; and (2) Representativeness: from these stable periods, 20 data points were selected to cover the observed range of key operational parameters (e.g., inlet temperatures and mass flow rates). This method ensures that the derived performance characteristics are robust and representative of the system’s behavior under the tested conditions. The primary measured parameters were as follows:

Air side: inlet and outlet moist-air dry-bulb temperatures, relative humidity, and inlet air mass flow rate; Solution side: inlet and outlet solution temperatures, solution mass flow rate, and circulating medium density;

Equipment power consumption: heat pump unit, fan, heat-source-side circulation pump, and user-side circulation pump.

**Fig 4:**
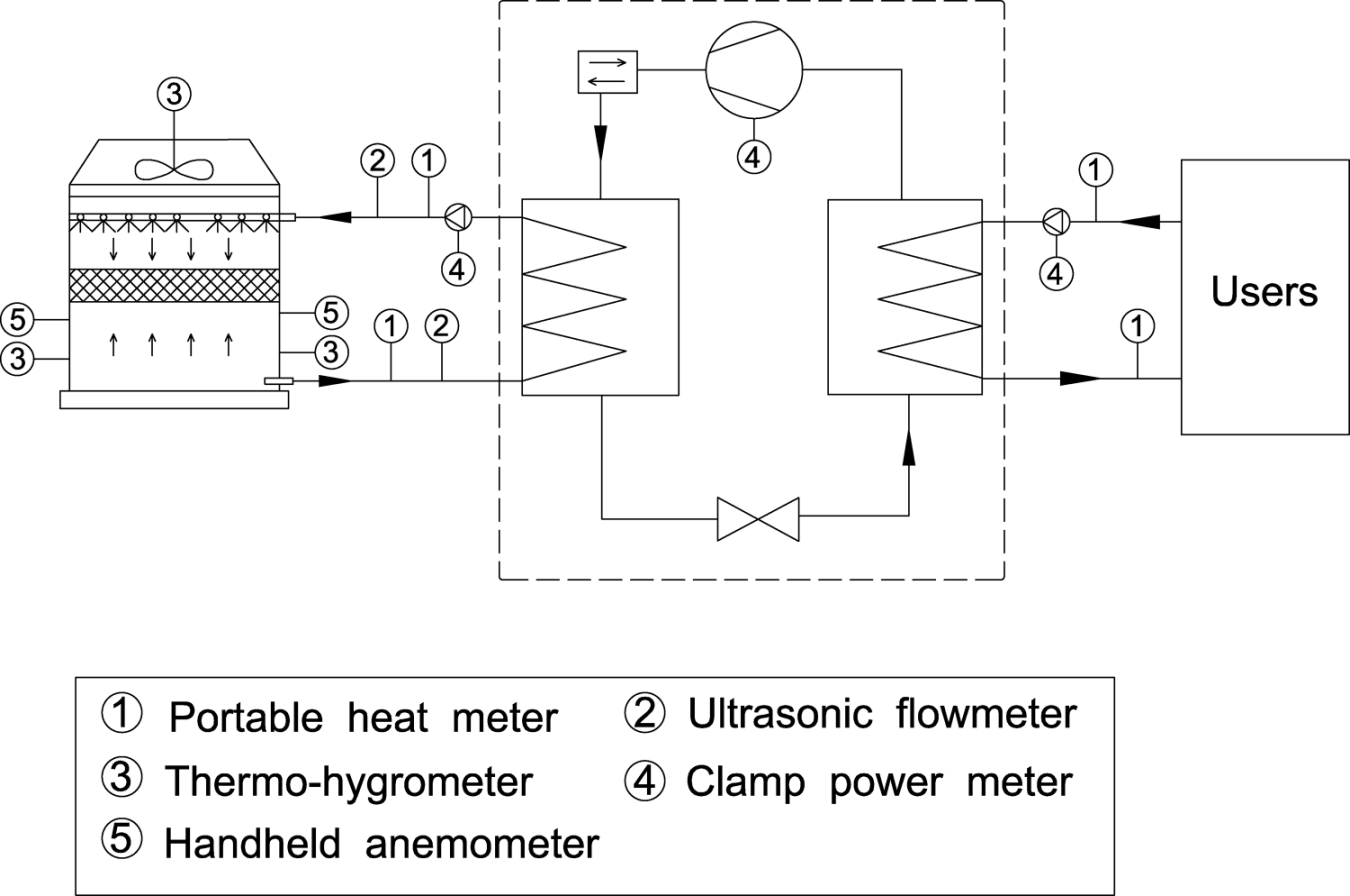
Measurement points layout.

Air-side and solution-side parameters were measured every 5 minutes, while equipment power was recorded every 15 minutes. The test parameters and the instruments employed are summarized in Table 2. The layout of all measurement points is illustrated in Fig 4.

**Table 2:** Measurement parameters and instruments.

Instrument	Type	Range	Parameter	Accuracy
Handheld anemometer	F9252111	0.40 ~ 25.00 m/s	Air flow rate	± 2%
Thermo-hygrometer	JR900	−10 ~ 50 °C, 0 ~ 100%	Air temperature, relative humidity	±1 °C, ± 5%
Ultrasonic flowmeter	TUF-2000P	0 ~ 32 m/s, -20 ~ 60 °C	Solution flow rate and temperature	± 1%
Portable heat meter	TUF-2000H	0 ~ 10 m/s, -30 ~ 90 °C		
Clamp power meter	PM2203	0.1 ~ 600 KW	Power consumption	± 3%

### 3.2. Error analysis

**Fig 5:**
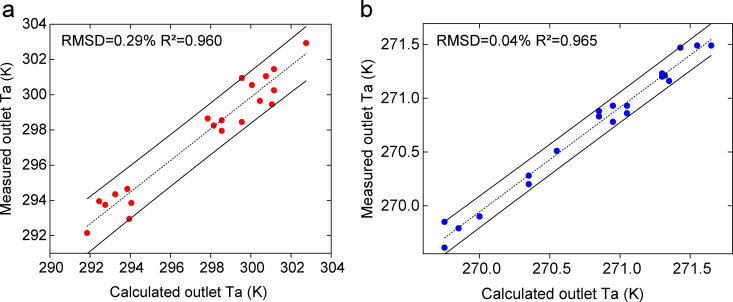
Comparison between measured and calculated outlet air temperatures under: (a) summer conditions. (b) winter conditions.

**Fig 6:**
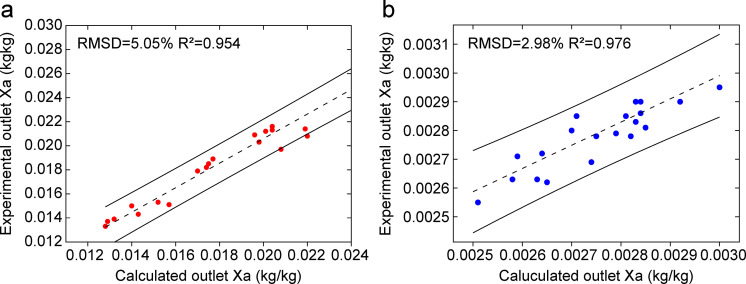
Comparison between measured and calculated outlet air humidity ratios under: (a).

**Fig 7:**
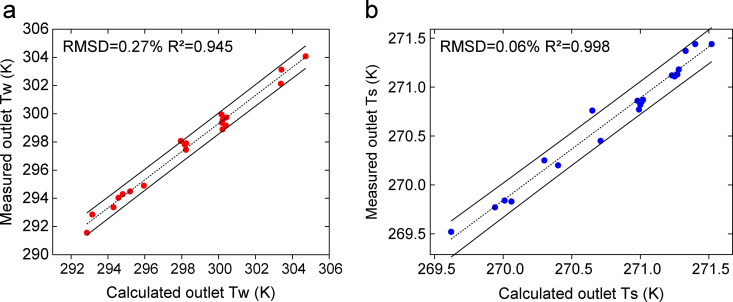
Comparison between measured and calculated outlet solution temperatures under: (a) summer conditions. (b) winter conditions.


Based on the measured air and solution data, the values were substituted into the summer and winter models for calculation. The outlet air temperature, humidity ratio, and solution temperature were obtained. Error analysis was performed using the root mean square deviation *RMSD* and the coefficient of determination *R²*. *RMSD* quantifies the average magnitude of the prediction errors for individual parameters, while *R²* evaluates the overall goodness of fit by indicating the proportion of variance in the measured data explained by the model. The results showed that the *RMSD* values were 0.29%, 5.05%, and 0.27% in summer; 0.04%, 2.98%, and 0.06% in winter. The corresponding *R*²values all exceeded 0.94, confirming a strong correlation between model predictions and experimental measurements. The comparison between the calculated and measured values is shown in Figs 5–7. The measured and calculated data used for model validation are provided in the Supporting Information (S1 File). The low *RMSD* values (all below 5.05%) combined with the high *R²* values (all above 0.94) confirm that the model can reliably predict the performance of open counterflow heat-source towers. It is essential to note that both *RMSD* and *R²* are applied only to the analysis of direct state parameters (temperature and humidity ratio); for derived quantities, such as the heat load and *COP*, error propagation methods were employed instead. An uncertainty propagation analysis conducted for the derived system performance metrics yielded results consistent with the *RMSD* and *R²* analyses, collectively confirming that the overall prediction errors of the model are within 10% for its key operating conditions.

$$RMSD=\sqrt{\frac{\text{1}}{N}\sum_{i=1}^{N}{\left(\frac{X_{ex}-X_{ca}}{X_{ca}}\right)}^{2}}$$ (35)

$$R^{2}=1-\frac{\sum {\mathrm{\backslash nolimits}}_{i=1}^{N}{\left(y_{i}-{\hat{y}}_{i}\right)}^{2}}{\sum {\mathrm{\backslash nolimits}}_{i=1}^{N}{\left(y_{i}-\bar{y}\right)}^{2}}$$ (36)

Where $X_{ex}$ is the measured value, $X_{ca}$ is the calculated value, $y_{i}$ is the i-th measured value, ${\hat{y}}_{i}$ is the i-th calculated value, $\bar{y}$ is the mean of all measured values, and *N* is the number of data samples. The temperature is based on the Kelvin scale, the unit for humidity ratio is kg/kg.

summer conditions. (b) winter conditions.

## 4. Heat transfer characteristics study

### 4.1. Performance evaluation indicators

Based on the national standard Code for Design of Heating, Ventilation, and Air Conditioning in Civil Buildings (GB50736−2012) issued by the Ministry of Housing and Urban-Rural Development [35], the summer ventilation design temperature for Guiyang is 27.1 °C with a relative humidity of 64%. For winter heating, the outdoor design temperature is −1 °C, while the ventilation design temperature is 5 °C with a relative humidity of 80%. These design parameters serve as representative boundary conditions for simulating the heat and mass transfer processes of the HTHP system in Guiyang’s high-humidity climate, forming the basis for subsequent heat transfer analysis.

To further investigate the heat transfer performance of open counterflow heat-source towers in Guiyang, the heat and mass transfer characteristics during summer and winter were evaluated in terms of sensible heat transfer, latent heat transfer, total heat transfer, and latent heat ratio.


$$Q_{s}=\left(C_{pa}+X_{a}\cdot C_{pv}\right)G_{da}\left(t_{a,o}-t_{a,i}\right)$$ (37)

$$Q_{l}=r_{o}\cdot G_{da}\cdot \left(X_{a,o}-X_{a,i}\right)$$ (38)

$$Q_{t}=Q_{s}+Q_{l}$$ (39)

$$\eta =Q_{l}/Q_{t}$$ (40)

Where $Q_{s}$ is the sensible heat transfer, $Q_{l}$ is the latent heat transfer, and is the total heat transfer, kW; η is the latent heat ratio, %.

Considering the influence of outdoor conditions on heat and mass transfer in year-round high-humidity regions, with Guiyang as a representative case, the parameter variation ranges were defined as listed in Tables 3 and 4.

**Table 3:** Summer simulation conditions.

Parameter	Inlet air temperature(°C)	Inlet water temperature(°C)	Inlet air humidity ratio (g/kg)	Inlet air mass flow rate (kg/s)	Inlet water mass flow rate (kg/s)
Inlet air temperature	22.0 ~ 28.0	30.0	13.8	50.0	67.0
Inlet water temperature	24.0	28.0 ~ 34.0	13.8	50.0	67.0
Inlet air humidity ratio	24.0	30.0	9.3 ~ 16.0	50.0	67.0
Inlet water mass flow rate	24.0	30.0	13.8	50.0	30.0 ~ 90.0

**Table 4:** Winter simulation conditions.

Parameter	Inlet air temperature(°C)	Inlet solution temperature (°C)	Inlet air humidity ratio (g/kg)	Inlet air mass flow rate (kg/s)	Inlet solution mass flow rate (kg/s)
Inlet air temperature	−1.0 ~ 7.0	−3.0	4.3	60.0	80.0
Inlet solution temperature	5.0	−5.0 ~ 3.0	4.3	60.0	80.0
Inlet air humidity ratio	5.0	−3.0	3.0 ~ 5.1	60.0	80.0
Inlet solution mass flow rate	5.0	−3.0	4.3	60.0	40.0 ~ 100.0

### 4.2. Effect of inlet solution temperature on heat and mass transfer

**Fig 8:**
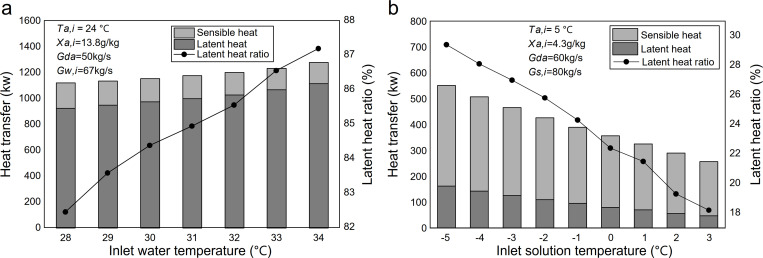
Effect of inlet solution temperature on heat transfer: (a) summer; (b) winter.

Fig 8(a) shows the effect of inlet water temperature on heat transfer in the heat-source tower during summer. As the inlet water temperature increased from 28.0 °C to 34.0 °C, the total heat transfer rose from 1124.2 kW to 1280.8 kW, an increase of 14.1%. Within this range, sensible heat transfer decreased from 197 kW to 164.1 kW, representing a 16.7% reduction, while latent heat transfer increased by 20.7%. At the same time, the proportion of latent heat transfer in the total heat transfer, i.e., the latent heat ratio, increased from 82.45% to 87.19%.

In heat and mass transfer theory, the exchange between water and air encompasses both sensible (heat) and latent (mass) transfer processes. The temperature difference between air and water is the driving force for sensible heat transfer. The mass transfer driving force is the water-vapor partial pressure difference between the air and the liquid surface (water-to-air for evaporation and air-to-solution for absorption). When air and water flow in opposite directions, a saturated air boundary layer forms at the water surface. As water temperature rises, the water vapor partial pressure and the air temperature within this boundary layer rise. Consequently, the increase in latent heat transfer is attributed to the higher water temperature, which enlarges the partial pressure difference of water vapor between air and water inside the heat-source tower. Simultaneously, enhanced vapor migration from water to air accelerates evaporation, allowing the air to absorb more latent heat. As evaporation becomes the dominant mode of heat transfer, the increase in air temperature is reduced, resulting in a smaller temperature difference between the water and air, and consequently, a decrease in sensible heat transfer. As the increase in latent heat outweighs the decrease in sensible heat, both the total heat transfer and the latent heat ratio rise.

Fig 8(b) illustrates the effect of varying inlet calcium chloride solution temperatures on heat transfer in the winter heat source tower. As the inlet solution temperature increased from −5.0 °C to 3.0 °C, the sensible heat transfer decreased from 388.58 kW to 209.61 kW, representing a 46.06% reduction. Latent heat transfer decreased from 162.06 kW to 46.52 kW, representing a 71.3% reduction, while total heat transfer dropped from 550.64 kW to 256.13 kW, a 53.5% decrease. Concurrently, the latent heat ratio decreased from 29.4% to 18.2%. The simultaneous decrease in sensible and latent heat transfer is attributed to the weakening of the temperature-difference driving force for heat transfer and the partial-pressure driving force for mass transfer of water vapor as the inlet solution temperature rises. This weakening also accounts for the marked decline in total heat transfer.

The opposite trends in Fig 8 can be understood by examining how the inlet fluid temperature affects the primary driving forces for heat and mass transfer in each season. In summer, the circulating medium is water. An increase in inlet water temperature raises the saturated vapor pressure at the water surface, significantly enlarging the partial pressure difference that drives evaporation, thereby strengthening latent heat transfer. Meanwhile, enhanced evaporation suppresses the air temperature rise within the tower, reducing the effective air–water temperature difference and thereby decreasing sensible heat transfer. In winter, the circulating medium is a hygroscopic calcium chloride solution that absorbs moisture. Increasing the inlet solution temperature has a dual weakening effect: it reduces both the air–solution temperature difference (sensible driving force) and the effective partial pressure difference for moisture absorption (latent driving force). This is because the vapor pressure at the solution surface is influenced by both its temperature and concentration (which may decrease due to dilution when regeneration is insufficient). Therefore, the distinct seasonal responses stem from the governing mechanisms: evaporation in summer is primarily driven by temperature, whereas absorption in winter is influenced by the combined effects of temperature and solution concentration, which together determine the surface vapor pressure.

Based on the above analysis, regulating the inlet solution temperature is critical for maintaining stable year-round performance of the heat-source tower. In winter absorption operation, lowering the solution temperature can enhance moisture uptake and improve latent heat transfer; however, excessive cooling may intensify moisture absorption and accelerate dilution (i.e., concentration reduction) when regeneration is insufficient, thereby increasing the solution freezing point and elevating the risk of icing. In our field operation, the inlet solution temperature is maintained at −3.0 °C to −1.0 °C to balance moisture absorption and freeze protection. For high-humidity winter conditions in Guiyang, incorporating a dedicated solution-regeneration unit is recommended to mitigate icing risk and sustain heat-exchange performance over extended operation.

### 4.3. Effect of inlet air humidity ratio on heat and mass transfer

**Fig 9:**
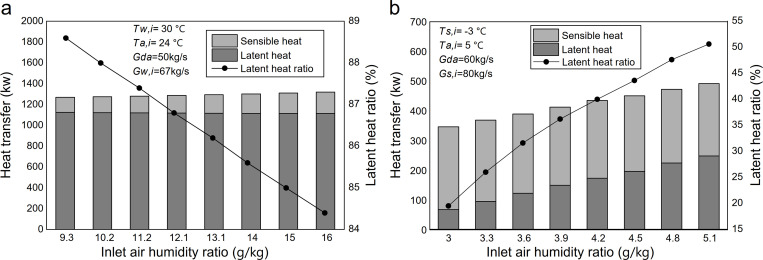
Effect of inlet air humidity ratio on heat transfer: (a) summer; (b) winter.

Fig 9(a) shows the effect of inlet air humidity ratio on heat transfer in the heat-source tower during the cooling season. As humidity rose from 9.3 g/kg to 16.0 g/kg, latent heat transfer remained nearly constant, dropping slightly from 1125.5 kW to 1113.8 kW (1%). In contrast, sensible heat transfer rose significantly from 144.3 kW to 205.2 kW, a 42.2% rise. Thus, total heat transfer rose from 1269.8 kW to 1319 kW, up 3.9%, while the latent heat ratio declined from 88.63% to 84.4%. The coupled heat-mass transfer mechanism can explain this behavior under evaporation-dominated summer operation. Increasing the inlet air humidity ratio raises the actual water-vapor partial pressure in the air, which would, in isolation, reduce the vapor/partial-pressure difference between the water surface and the air and suppress latent heat transfer. The observed minimal (1%) reduction in latent heat transfer, however, indicates that this suppressing effect was largely counteracted within the tested system. This can be attributed to the operating conditions: the maintained warm inlet water temperature (30 °C) sustains a high saturated vapor pressure at the water surface, thereby partially offsetting the increase in air-side vapor partial pressure. Consequently, the effective net driving force for mass transfer remained relatively stable, limiting the variation in the latent component. Meanwhile, suppressed evaporation weakens evaporative cooling, shifting a larger fraction of the heat exchange toward sensible heating of the air, which leads to higher sensible heat transfer and a lower latent heat ratio. Overall, variations in inlet air humidity ratio primarily redistribute latent versus sensible contributions rather than significantly changing the total heat transfer.

Fig 9(b) illustrates the effect of varying inlet air humidity ratio on heat transfer in the heat source tower during the heating season. As the humidity ratio increased from 3.0 g/kg to 5.1 g/kg, sensible heat transfer decreased from 278.78 kW to 243.09 kW, a 12.8% reduction. In contrast, latent heat transfer increased substantially from 67.53 kW to 249.1 kW, while total heat transfer rose from 346.31 kW to 492.19 kW, representing a 42.2% increase. The significant increase in latent heat transfer is attributed to the rising humidity ratio, which elevates the vapor partial pressure in the mainstream air, thereby enlarging the partial-pressure driving force for moisture absorption (i.e., the water-vapor partial-pressure difference between the mainstream air and the calcium chloride solution surface). This pronounced increase in vapor pressure difference promotes the condensation of water vapor at the interface and accelerates its absorption into the solution, thereby enhancing moisture absorption. The reduction in sensible heat transfer occurs because part of the latent heat warms the boundary layer air, while most of the heat is transferred to the solution, raising its temperature. However, the temperature drop of the bulk air is relatively minor, reducing the temperature difference between the bulk air and the solution, weakening the driving force for sensible heat transfer. Hence, maintaining inlet air humidity within a moderate range helps stabilize heat transfer performance. This is particularly important for ensuring reliable heating operation under high-humidity winter conditions.

### 4.4. Effect of inlet air temperature on heat and mass transfer

**Fig 10:**
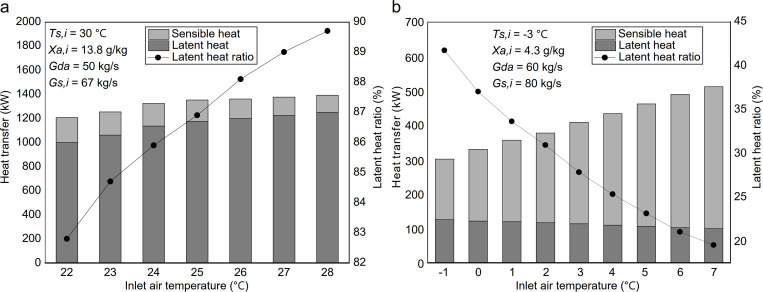
Effect of inlet air temperature on heat transfer: (a) summer; (b) winter.

Fig 10(a) presents the heat-source tower’s heat transfer performance in summer under different inlet air temperatures. As the inlet air temperature increases from 22.0 °C to 28.0 °C, total heat transfer rises from 1207.5 kW to 1393.3 kW, a 15% increase. Sensible heat transfer decreases from 207.1 kW to 142.84 kW, a reduction of 31%, while latent heat transfer increases from 1000.4 kW to 1250.5 kW, an increase of 25%. The latent heat ratio gradually increases, though at a diminishing rate. This trend is governed by the coupled sensible–latent heat transfer characteristic of evaporation-dominated summer operation. As the inlet air temperature increases, the air–water temperature difference decreases, directly weakening the driving force for sensible heat transfer and accounting for the observed reduction in sensible heat. In parallel, although warmer inlet air elevates the actual water-vapor partial pressure in the air, this increase is outpaced by the rise in the saturated vapor pressure at the water surface under the maintained warm water conditions. Consequently, the vapor pressure difference between air and water that drives evaporation increases, thereby enhancing latent heat transfer. The diminishing growth rate of the latent heat ratio suggests that, at higher air temperatures, further increases in evaporation become less pronounced, resulting in progressively smaller gains in latent heat transfer per unit temperature increase.

Fig 10(b) presents the heat-source tower’s heat transfer performance in winter under varying inlet air temperatures. As the inlet air temperature increases from −1.0 °C to 7.0 °C, total heat transfer rises from 301.86 kW to 511.94 kW, a 69.6% increase. Sensible heat transfer increases markedly from 176.11 kW to 412 kW, while latent heat transfer decreases from 125.75 kW to 99.94 kW, a reduction of 20.53%. Consequently, the proportion of latent heat transfer in total heat transfer gradually decreases. During winter, the solution temperature remains relatively low. As the inlet air temperature rises, the temperature difference between the solution and the air increases, accounting for the significant enhancement in sensible heat transfer. Although the inlet air temperature increases, the humidity ratio remains nearly constant, so the actual water-vapor partial pressure in the mainstream air changes only slightly. Accordingly, the mass-transfer driving force for moisture absorption, expressed as the water-vapor partial-pressure difference between the mainstream air and the solution surface, decreases only marginally. Meanwhile, the solution temperature increases only slightly, so the water-vapor pressure at the solution surface rises slowly, leading to only a minor reduction in latent heat transfer. In practical applications, maintaining a moderate inlet air temperature is beneficial for enhancing sensible heat transfer and ensuring stable heating performance during winter operation.

### 4.5. Effect of inlet solution mass flow rate on heat and mass transfer

**Fig 11:**
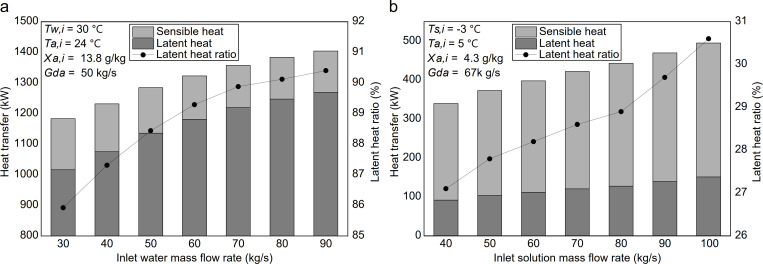
Effect of inlet solution mass flow rate on heat transfer: (a) summer; (b) winter.

Fig 11(a) illustrates the variation of heat transfer in the summer heat-source tower under different inlet water mass flow rates. It can be observed that as the inlet water flow rate increases from 30.0 kg/s to 90.0 kg/s, the total heat transfer increases from 1183.2 kW to 1404.3 kW (an 18.7% increase), and the sensible heat transfer decreases from 166.7 kW to 135.2 kW (an 18.9% decrease). In contrast, the latent heat transfer increases from 1016.5 kW to 1269.1 kW (a 24.8% increase). Meanwhile, the latent heat ratio exhibits a slight rise of 4.5%. This behavior reflects the coupled heat and mass transfer dynamics under evaporation-dominated summer operation. As the inlet water mass flow rate increases, the temperature change of water per unit mass decreases, leading to a more uniform temperature profile within the packing that approaches the relatively high inlet value. This condition influences the two heat-transfer pathways differently. On one hand, the maintained higher water-surface temperature keeps a high saturated vapor pressure at the water-air interface. Since the air-side vapor pressure is relatively insensitive to flow variation, the vapor-pressure difference driving evaporation is preserved or may slightly increase, enhancing latent heat transfer. On the other hand, the reduced effective air–water temperature difference weakens the driving force for sensible heat transfer. Furthermore, the intensified evaporation consumes a larger fraction of the exchanged energy as latent heat, thereby further suppressing the air temperature rise and reducing sensible heat transfer. Consequently, the gain in latent heat exceeds the loss in sensible heat, resulting in higher total heat transfer and a slightly higher latent heat ratio.

Fig 11(b) illustrates the variation of heat transfer in the heat-source tower during winter with different inlet solution mass flow rates. It can be observed that as the inlet solution flow rate increases from 40.0 kg/s to 100.0 kg/s, the total heat transfer rises from 340 kW to 495 kW (a 45.6% increase), sensible heat transfer rises from 247.9 kW to 343.5 kW (a 38.6% increase), and latent heat transfer rises from 92.1 kW to 151.5 kW (a 64.5% increase). In contrast, the latent heat ratio increases slightly by only 3.5%. While this behavior shares some principles with summer operation, key differences arise in winter due to the lower solution temperature and the consequent larger air-solution temperature difference. As the inlet solution mass flow rate increases, the effective air-solution temperature difference increases, which accounts for the observed rise in sensible heat transfer. In contrast to summer, the air temperature in winter is higher than the solution temperature, so the temperature gradient between air and solution strengthens, enhancing sensible heat transfer. The increase in sensible heat transfer is the dominant contributor to the overall increase in total heat transfer. Meanwhile, although the increased flow rate reduces the temperature rise of the solution along the tower and the outlet temperature approaches the inlet temperature, the solution’s relatively low temperature and the limited increase in the saturated vapor pressure at the solution surface result in only a slight increase in latent heat transfer. The effect of increased flow rate on the partial-pressure difference for moisture absorption is more limited in winter, since the actual water-vapor partial pressure in the air remains low due to the cooler air temperatures. As a result, the solution’s moisture absorption is initially enhanced. Then it tends to stabilize, leading to a more modest increase in latent heat transfer compared to the increase in sensible heat transfer.

From an engineering perspective, adjusting the inlet solution mass flow rate within an appropriate range helps balance water pump energy consumption and total heat transfer during seasonal operation. This parameter also indirectly affects the total system input power discussed in the following section, which analyzes the variation of the system’s *COP* under typical summer and winter conditions and throughout the cooling and heating seasons. The detailed datasets corresponding to the variations of latent heat, sensible heat, and latent heat ratio with inlet solution temperature, solution mass flow rate, air temperature, and air humidity ratio are provided in the Supporting Information (S2 File).

## 5. Year-round performance analysis of the HTHP system

### 5.1. Performance metrics

Based on the cooling and heating seasons, the annual operational performance of the HTHP system under high-humidity conditions was comprehensively assessed using the following metrics: Coefficient of Performance (*COP*), Theoretical Coefficient of Performance (*COP*_*t*_), Seasonal Energy Efficiency Ratio (*SEER*), Heating Seasonal Performance Factor (*HSPF*), and Annual Performance Factor (*APF*). These metrics collectively evaluate the system’s efficiency from instantaneous, seasonal, and annual perspectives.

The theoretical coefficient of performance (*COP*_*t*_), based on the Carnot cycle, represents the upper efficiency limit under ideal reversible conditions. In contrast, the actual *COP*, defined by Equations (41) and (42), is significantly lower. The underlying physical mechanism for this discrepancy lies in the irreversible losses during actual operation (e.g., heat transfer temperature differences, flow resistance, and compressor inefficiency). These losses directly or indirectly cause the operating evaporating temperature to be lower and the condensing temperature to be higher than their ideal values. This increases the compressor pressure ratio, leading to a marked rise in compressor power consumption and, consequently, a lower actual *COP*. Therefore, the analysis of the actual *COP* focuses on how external parameters and operating conditions influence system performance by affecting the evaporating and condensing temperatures. Sections 5.2 and 5.3 will specifically analyze the impact of outdoor temperature and humidity on *COP* through this pathway under typical operating conditions, followed by a comparison between *COP* and *COP*_*t*_ and an evaluation of annual energy efficiency in Section 5.4.

The summer cooling *COP*_*c*_ test was conducted from June 15–19, 2023, and the winter heating *COP*_*h*_ test from January 5–9, 2024, under Guiyang’s typical high-humidity climate conditions. *SEER* was evaluated from May 24 to September 20, 2023, covering an entire cooling season, while *HSPF* was assessed from November 17, 2023, to March 15, 2024, spanning a complete heating season.


$$COP_{C}=\frac{Q_{C}}{E_{hp}+E_{f}+E_{S}+E_{l}}$$ (41)

$$COP_{h}=\frac{Q_{h}}{E_{hp}+E_{f}+E_{S}+E_{l}}$$ (42)

$$COP_{ct}=\frac{t_{evap}}{t_{cond}-t_{evap}}$$ (43)

$$COP_{ht}=\frac{t_{cond}}{t_{cond}-t_{evap}}$$ (44)

$$Q_{c}=m_{chw}\cdot c_{p,chw}\left(t_{chw,i}-t_{chw,o}\right)$$ (45)

$$Q_{h}=m_{hw}\cdot c_{p,hw}\left(t_{hw,o}-t_{hw,i}\right)$$ (46)

Where $\;Q_{C}\;$ is the cooling capacity supplied to the user side during summer, and $Q_{h}$ is the heating capacity provided to the user side during winter. $E_{hp}$ is the input power of the heat pump unit, $E_{l}$ the power consumption of the user-side water pump, $E_{s}$ the power consumption of the source-side water pump, and the power consumption of the heat-source tower fan, all in kW, $t_{cond}\;$ and $t_{evap}$ denote the condensing and evaporating temperatures, respectively, in K. The subscripts chw and hw represent chilled water and hot water, respectively. The equipment parameters of the HTHP system are listed in Table 5, which reflects the actual configuration adopted in the experimental setup and ensures representativeness for engineering-scale operation.

**Table 5:** System equipment.

Name	Quantity	Type	Parameter
Heat pump unit	3	11100S/L-R2(R134a)	Power (Cooling):197kW Power (Heating):233kW
Heat-source tower	3	XPS-E9S	Power:15kW
User-side pump	4	DFWH150-400B/4/30	Power:30 kw
Source-side pump	4	DFWH150-400A/4/37	Power:37kW


$$SEER=\frac{\sum Q_{c}}{\sum W_{c}}$$ (47)

$$HSPF=\frac{\sum Q_{h}}{\sum W_{h}}$$ (48)

$$APF=\frac{\sum Q_{c}+\sum Q_{h}}{\sum W_{c}+\sum W_{h}}$$ (49)

$$\Sigma W=\sum_{i=1}^{N-1}\frac{P_{i}+P_{i+1}}{\text{2}}\cdot \Delta t_{i}$$ (50)

Where $\sum Q_{c}$ is the cumulative cooling capacity in summer, $\sum Q_{h}$ is the cumulative heating capacity in winter, $\sum W_{c}$ is the cumulative electricity consumption in summer, and $\;\sum W_{h}\;$ is the cumulative electricity consumption in winter, all expressed in kW·h; $P_{i}$ and $P_{i+1}$ are the system’s total instantaneous power (the denominator of the formulas 41 and 42) at two adjacent measurement points, kW; $\Delta t_{i}$ is the constant measurement interval (0.25 hours); *N* is the total number of data points.

The system operates for 8 hours daily (09:00–17:00) to meet the cooling and heating demands of the office building during working hours, and remains shut down outside this period. Therefore, the daily energy consumption can be reasonably estimated by integrating the measured power over this core operational interval. The seasonal cumulative energy consumption is then obtained by summing the estimated daily values over all operating days. This approach ensures that the calculated *SEER*, *HSPF*, and *APF* are based on a representative energy consumption profile, accurately reflecting the system’s primary operating pattern.

### 5.2. Energy efficiency analysis under typical cooling season conditions

**Fig 12:**
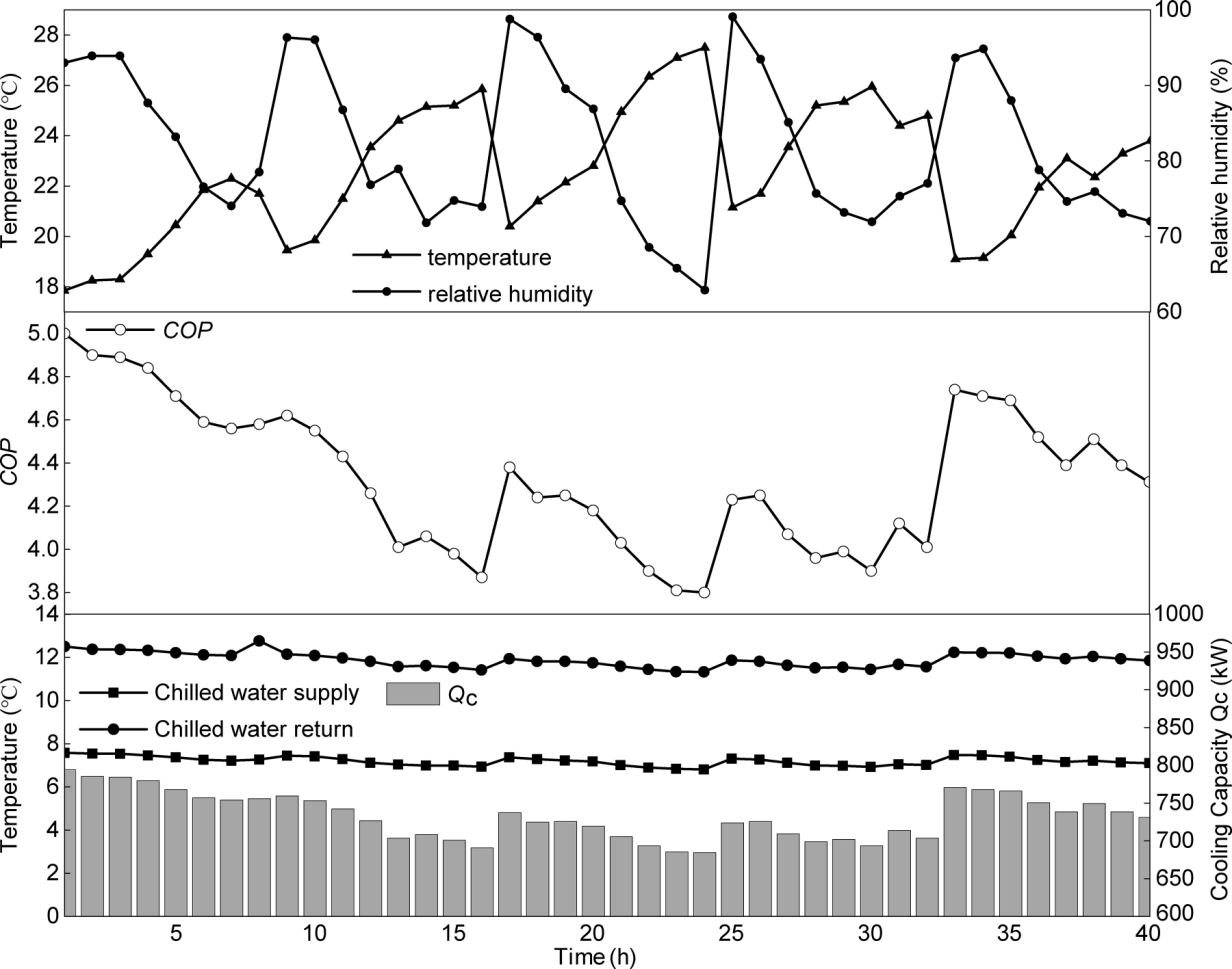
Temporal variations of air temperature and relative humidity, chilled water temperature, cooling capacity, and *COP.*

Fig 12 represents the continuous variations in outdoor air temperature and relative humidity, chilled water supply and return temperatures, cooling capacity, and *COP* of the heat pump system from June 15–19, 2023. Despite fluctuations in outdoor meteorological conditions, the chilled water supply and return temperatures on the evaporator side remained relatively stable, with average values of 7.2°C and 11.87 °C, respectively. The heat pump unit delivered an average cooling capacity of 733.7 kW. Overall, the *COP* exhibited a negative correlation with outdoor air temperature and a positive correlation with relative humidity. The maximum *COP* was observed during the first hour of testing, coinciding with a period immediately after rainfall in Guiyang, when the outdoor air temperature was at its lowest and the relative humidity was relatively high. At this time, the outlet water temperature of the heat-source tower decreased due to the lower air wet-bulb temperature, which reduced the condensing temperature. Consequently, the compressor pressure ratio declined, lowering the compressor’s input power. The figure shows that the system’s cooling capacity reached its maximum, while the total power consumption was relatively low. According to Equation (41), these factors explain why the *COP* peaked at 5 during this period.

The lowest *COP* was recorded at 2:00 PM on June 17 (the 24th hour of testing), when the outdoor air temperature peaked, and relative humidity was at its minimum. The minimum *COP* of 3.8 during this period can be attributed to the rise in air temperature and the drop in relative humidity. Both the sensible and latent heat driving forces within the heat source tower weakened, resulting in a reduction in total heat transfer. The elevated outlet water temperature of the tower caused the condensing temperature to rise, resulting in an increased compressor pressure ratio and input power. Meanwhile, heat transfer in the evaporator was restricted, and the reduced refrigerant circulation flow resulted in a decline in cooling capacity.

### 5.3. Energy efficiency analysis under typical heating season conditions

**Fig 13:**
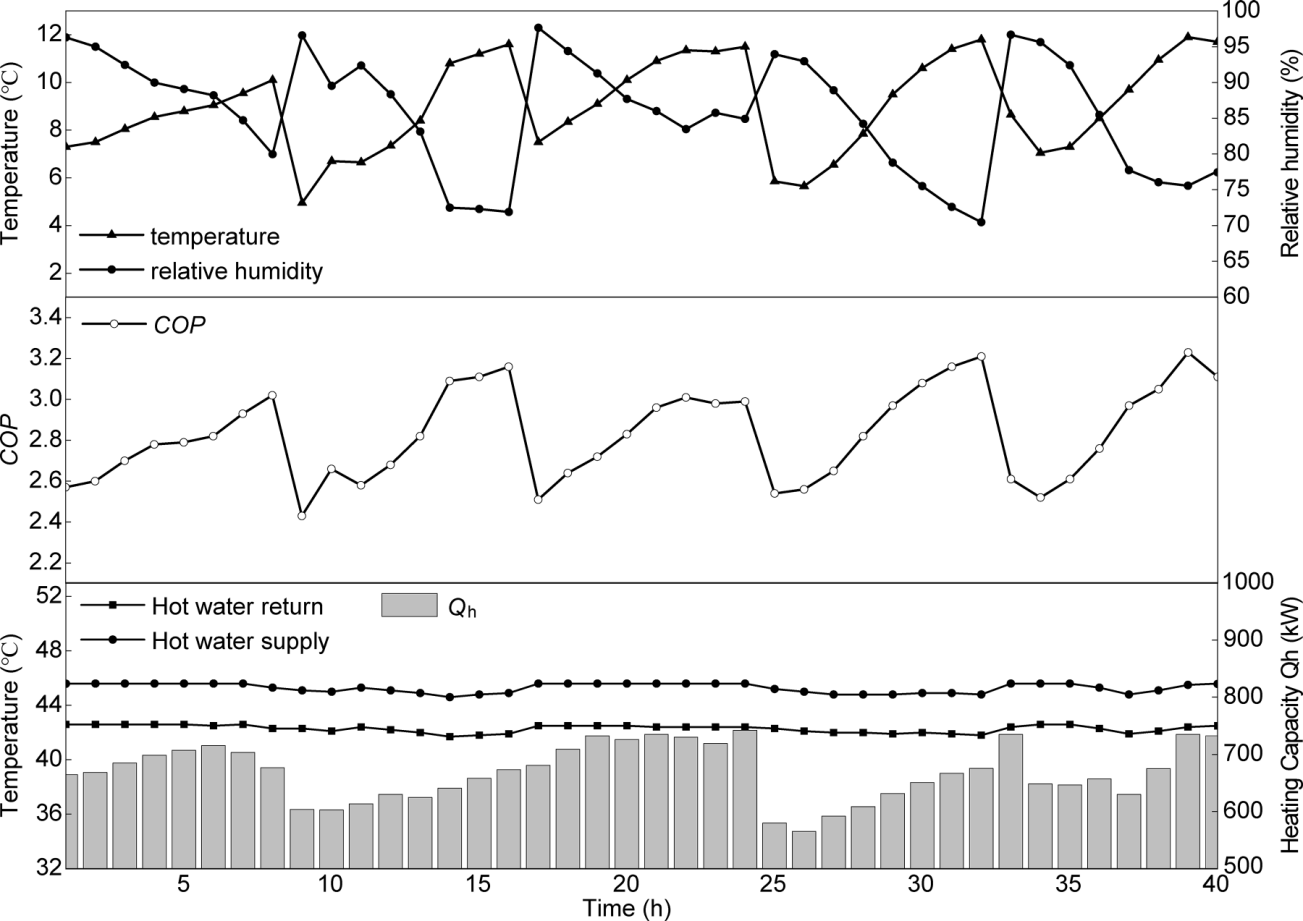
Temporal variations of air temperature and relative humidity, hot water temperature, heating capacity, and *COP.*

Fig 13 presents the continuous variations in outdoor air temperature and relative humidity, hot water supply and return temperatures, heating capacity, and *COP* of the heat pump system from January 5–9, 2024. Despite fluctuations in outdoor meteorological conditions, the hot water supply and return temperatures on the condenser side remained relatively stable, with average values of 45.1 °C and 42.2 °C, respectively. The heat pump unit delivered an average heating capacity of 675 kW. During the heating season, in contrast to the cooling season, the *COP* increased with rising outdoor air temperature and decreased with higher relative humidity. The maximum *COP* was observed at the 39th hour of testing (2:00 PM on January 9), when the outdoor air temperature was highest and the relative humidity was relatively low. At this time, enhanced sensible and latent heat transfer led to the maximum total heat transfer. As a result, the evaporating temperature increased, which lowered the compressor pressure ratio, reduced the compressor’s input power, and decreased the total system power consumption. According to Equation (42), these factors explain why the *COP* reached its peak value of 3.23 during this period. The minimum *COP* was recorded at the 9th hour of testing (8:00 AM on January 6), when the outdoor air temperature was lowest and the relative humidity was relatively high. At this time, the *COP* dropped to 2.43 due to the combined effects of a low wet-bulb temperature, which reduced the sensible heat transfer capacity, and a smaller vapor pressure difference, which limited latent heat transfer. These factors significantly reduced total heat transfer, resulting in a lower evaporating temperature, an increased compressor pressure ratio, and higher power consumption.

Based on the analysis presented in Sections 5.2 and 5.3, from a practical engineering perspective, maintaining appropriate condensing and evaporating temperatures is essential for the stable and efficient operation of the HTHP system. The system should effectively prevent excessively high condensing temperatures or overly low evaporating temperatures during cooling operations, as well as excessively low evaporating temperatures or high condensing temperatures during heating operations. Therefore, the solution and air mass flow rates should be reasonably adjusted according to variations in outdoor temperature and humidity ratio, ensuring that the condensing and evaporating temperatures remain within an appropriate range. Such dynamic regulation enhances the system’s overall energy efficiency and operational reliability. The detailed operational data for both cooling and heating seasons, including temporal variations of air temperature, relative humidity, water temperature, capacity, and *COP*, are provided in the Supporting Information (S3 File).

### 5.4. Year-round energy efficiency analysis

**Fig 14:**
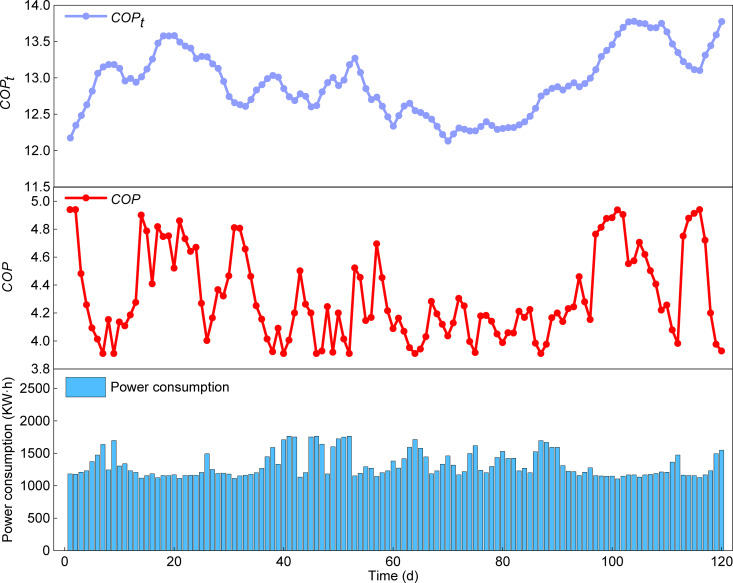
Temporal variations of total system energy consumption, *COP*, and theoretical *COP*_*t*_ in the cooling season.

**Fig 15:**
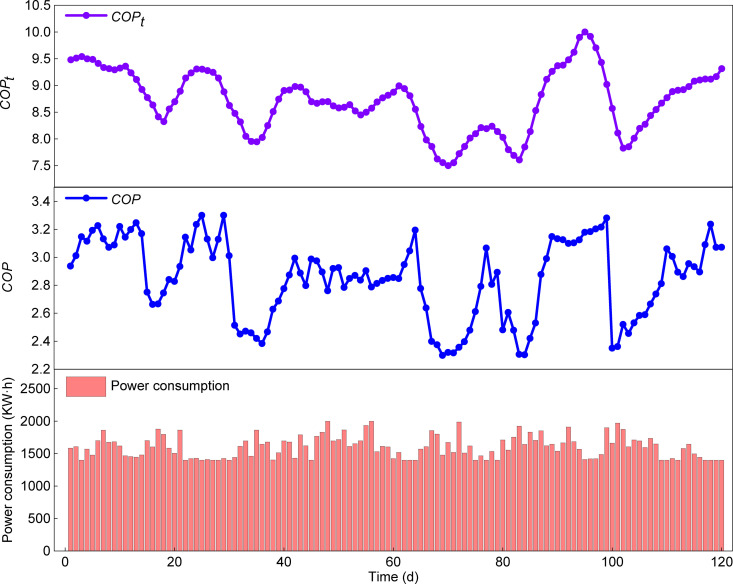
Temporal variations of total system energy consumption, *COP*, and theoretical *COP*_*t*_ in the heating season.

Building upon the analysis of typical operating conditions in June and January, which examined the temporal variation of *COP*, the subsequent analysis focuses on the seasonal and annual energy performance of the HTHP system. Figs 14 and 15 show the variations in total system energy consumption, *COP*, and theoretical *COP*_*t*_ over the cooling and heating seasons. Equations (43) and (44) indicate that the theoretical *COP*_*t*_ is determined by the condensing and evaporating temperatures, reflecting the maximum efficiency attainable under an ideal reverse Carnot cycle. By comparison, the *COP* depends on compressor efficiency and component losses, and is below *COP*_*t*_, representing the real engineering efficiency. Therefore, the role of *COP*_*t*_ is not to directly predict the actual *COP* but to serve as a critical reference framework. Its value and smooth trend provide an idealized baseline. The discrepancy between the *COP* and *COP*_*t*_ curves reflects the combined impact of all irreversible losses and the power consumption of auxiliary equipment (e.g., water pumps, fans) under actual operating conditions. Calculations from Equations (47), (48), and (49) yield a *SEER* of 4.3 for cooling, a *HSPF* of 3.06 for heating, and an *APF* of 3.62. This result exceeds the minimum performance coefficient requirements specified in GB50189−2015 Public Building Energy Efficiency Design Standard (cooling≥4.0; heating≥2.9) [36]. These results indicate that the HTHP system offers significant advantages in summer cooling and winter heating under typical high-humidity climatic conditions, delivering outstanding comprehensive energy efficiency performance throughout the year. The detailed datasets of total system energy consumption, *COP*, and theoretical *COP*_*t*_ for both cooling and heating seasons are provided in the Supporting Information (S4 File).

## 6. Conclusions

This study contributes to bridging the gap in year-round performance metrics for open-counterflow HTHP systems in high-humidity climates. An integrated experimental and numerical approach, based on typical seasonal conditions, was employed to develop and validate heat and mass transfer models for both summer and winter conditions. These models facilitated a systematic analysis of the effects of key inlet parameters, including solution temperature, air temperature, air humidity ratio, and solution mass flow rate. Finally, based on typical cooling and heating seasons, the year-round energy efficiency was evaluated through an engineering case study. Thus, this study delivers validated models, clarifies the distinct seasonal heat transfer mechanisms, and establishes annual performance metrics (*SEER*, *HSPF*, *APF*) for high-humidity climates. The key findings can be summarized as follows:

(1) : The heat transfer mechanism of the heat-source tower exhibits pronounced seasonal differences. Under summer conditions, water evaporates into the air, and latent heat transfer dominates heat transfer. In contrast, under winter conditions, the antifreeze solution absorbs moisture from the air, and heat transfer is primarily governed by sensible heat transfer. The proportion of latent heat transfer in the total heat transfer ranges from 82.45% to 90.4% in summer and from 16.3% to 50.6% in winter.
(2) : Under summer conditions, as the inlet air temperature increases from 22.0 °C to 28.0 °C, latent heat transfer rises by 25%, whereas sensible heat transfer decreases by 31%. The influence of the inlet air humidity ratio on sensible heat transfer is considerably greater than on latent heat transfer. When the inlet water temperature increases from 28.0 °C to 34.0 °C, latent heat transfer increases by 20.7%, sensible heat transfer decreases by 16.7%, and the latent heat ratio rises from 82.45% to 87.19%. As the inlet water mass flow rate increases from 30.0 kg/s to 90.0 kg/s, total heat transfer rises by 18.7%, with a slight 4.5% increase in the latent heat ratio.
(3) : During winter, sensible heat transfer increases substantially as the inlet air temperature rises from −1.0 °C to 7.0 °C. In contrast, latent heat transfer decreases by 20.53%, leading to a continuous decline in the latent heat ratio. The influence of inlet air humidity ratio on latent heat transfer is markedly greater than on sensible heat transfer, resulting in a continuous increase in the latent heat ratio. Sensible and latent heat transfer decreases significantly when the inlet calcium chloride solution temperature increases from −5.0 °C to 3.0 °C, and the latent heat ratio drops from 29.4% to 18.2%. As the inlet solution mass flow rate increases from 40.0 kg/s to 100.0 kg/s, total heat transfer rises by 45.6%, while the latent heat ratio slightly increases by 3.5%.
(4) : Under typical cooling season conditions (dry-bulb air temperatures 17.9–27.5°C; relative humidity 63–99%), the *COP* ranged from 3.8 to 5. Across the entire cooling season, the Seasonal Energy Efficiency Ratio (*SEER*) was calculated as 4.3. Under typical heating season conditions (dry-bulb air temperatures 5–11.9 °C; relative humidity 70–98%), the *COP* ranged from 2.43 to 3.23, and the Heating Seasonal Performance Factor (*HSPF*) was 3.06. Finally, the Annual Performance Factor (*APF*) of the HTHP system was 3.62, confirming its favorable year-round performance.

These results provide technical guidance for optimizing the design and operation of HTHP systems in regions with year-round high-humidity climates.

## Supporting information

S1 File Comparative datasets of measured and calculated outlet parameters, including solution temperature, air temperature, and air humidity ratio under summer and winter conditions. (XLSX)

S2 File Experimental datasets corresponding to the variations of latent heat, sensible heat, and latent heat ratio with inlet solution temperature, solution mass flow rate, air temperature, and air humidity ratio. (XLSX)

S3 File Datasets of typical operating conditions in summer and winter, including air temperature, relative humidity, water temperature, capacity, and *COP.* (XLSX)

S4 File Total system energy consumption, *COP*, and theoretical *COPₜ* data for both cooling and heating seasons. (XLSX)

S5 File MATLAB models for the summer and winter operating conditions of the heat-source tower heat pump system (included within *Model.zip*). (RAR)
